# Hypertension among Young Adults with Ischemic Stroke of a Tertiary Care Centre in Nepal: A Descriptive Cross-sectional Study

**DOI:** 10.31729/jnma.6812

**Published:** 2021-08-31

**Authors:** Dilli Ram Kafle, Sudhakar Jha

**Affiliations:** 1 Institute of Neuroscience, Nobel Medical College, Biratnagar, Nepal; 2 Department of Internal Medicine, Nobel Medical College, Biratnagar, Nepal

**Keywords:** *hypertension*, *stroke*, *transient ischemic attack*

## Abstract

**Introduction:**

Ischemic strokes in young patients have been increasing. Younger patients with ischemic stroke tend to have a different long-term prognosis than older patients. Young patients who have residual neurological deficits following ischemic stroke affect their quality of life. This study was carried out to find out the prevalence of hypertension among patients with ischemic stroke in a tertiary care hospital.

**Methods:**

This descriptive cross-sectional study was conducted in all the patients who were admitted with young ischemic stroke in the Department of Neurology, Nobel Medical College Biratnagar from December 2019 to December 2020 after taking ethical clearance from the Institutional Review Committee (reference number: 332/2019). Convenience sampling was done and data was collected, entered in Microsoft Excel, and analysis was done using Statistical Package for the Social Science software version 16. Point estimate at 95% Confidence Interval was calculated along with frequency and proportion for binary data.

**Results:**

Out of the total patients with ischemic stroke in the young adults 30 (40%) (95% Confidence Interval= 28.91-51.08) had hypertension. During follow-up at 6 months, 20 (66.7%) of the patients with hypertension had favorable outcomes with a Modified Rankin Score of 0 or 1. Death during a hospital stay or during follow-up was observed in 2 (7%) of patients with hypertension. Mean age of the patients was 40±4.87 years. Most patients were in the age range of 36-45, 24 (80%).

**Conclusions:**

Prevalence of hypertension among young patients with ischemic stroke was high compared to other studies.

## INTRODUCTION

Stroke is considered an important cause of morbidity and death worldwide. Each year, ischemic stroke occurs in more than 11 million patients.^1^ Earlier stroke was considered a disease of the older population. However, recent studies have shown that 10 to 15% of the total number of strokes occurs in younger patients.^2^ From 1980 to 2010, it has been reported that the incidence of ischemic stroke in young adults has been increasing worldwide, with a more pronounced increase of incidence in women.^3^

The causes of ischemic stroke in young adults are many and it tends to vary with age and geographic area. The TOAST (Trial of Org 10172 in Acute Stroke Treatment) criteria for the classification of acute ischemic stroke is most commonly used.^4^

The main aim of this study was to find out the prevalence of hypertension among patients with ischemic stroke in a tertiary care centre.

## METHODS

This is a descriptive cross-sectional study carried out at the department of neurology, Nobel Medical College, Biratnagar among young adults with ischemic stroke after obtaining ethical clearance from the Institutional Review Committee of Nobel Medical College (reference number: 332/2019). We enrolled all consecutive patients with ischemic stroke aged between 16 and 45 admitted in the neurology ward and intensive care unit of Nobel Medical College, Biratnagar between December 2019 and December 2020. Patients less than 16 and older than 45 years of age were excluded from the study. Similarly, patients who did not give consent for the study and those with missing data and incomplete information were excluded from the study. Ethical clearance for the study was obtained from the institutional review committee. The age 45 was taken as the upper limit to define ischemic stroke in young.^5^ Convenience sampling was done and the sample size was calculated using the formula,

n = Z^2^ × p × q / e^2^

  = (1.96)^2^ × (0.5) × (1-0.5) / (0.12)^2^

  = 67

Where,

n = minimum required sample size
Z = 1.96 at 95% Confidence Interval (CI)
p = prevalence for maximum sample size, 50%
q = 1-p
e = margin of error, 12%

Taking a 10% non-response rate, the calculated sample size is 74. We have taken 75 participants in the study.

Information was collected from the patient and their family members during the hospital stay. Demographic profiles including age, sex, the onset of stroke, clinical history, examination findings were recorded. The severity of stroke was assessed using the NIHSS scale with a higher score representing greater severity.^6^ Based on NIHSS, stroke severity was classified as mild (NIHSS score 0 to 6), moderate (7 to 15), or severe (>15). GCS was also documented. Similarly, etiology was classified according to TOAST criteria taking into consideration the finding of neuroimaging (CT/MRI) which was done in all patients. Blood investigations including complete blood cell count, blood sugar, renal function, electrolyte and workup for vasculitis were carried out in all patients. Electrocardiography, echocardiography, Doppler ultrasound of neck vessels was also done in all patients.

The six-month functional outcome was assessed using the Modified Rankin Scale (mRS) score.^7^ The mRS score was used to represent functional outcomes as favorable (score 0-1) or Unfavorable (score 2-6). Statistical analysis was done with Statistical Package for the Social Sciences (SPSS) software. Mean, standard deviation, frequencies and percentages were used to present descriptive statistics. Point estimate at 95% Confidence Interval was calculated along with frequency and proportion for binary data.

## RESULTS

Out of the total patients with ischemic stroke in the young adults, hypertension was found in 30 (40%) (28.91-51.08 at 95% Confidence Interval). During follow-up at 6 months, 20 (66.7%) of the patients with hypertension had favorable outcomes with mRS score of 0 or 1. Death during the hospital stay or during follow-up was observed in 2 (7%) of patients with hypertension. The mean age of the patients with hypertension was 40±4.87years. Most patients with hypertension were in the age range of 36-45 (80%). Duration of ischemic stroke before presenting to the hospital was 2.68±0.94 days while the mean duration of hospital stay was 5.74±6.07 days. Similarly, mean NIHSS on presentation to hospital was 9.28±6.84 while GCS at admission was 13.8±2.11 (Table 1).

**Table 1:** Clinical characteristics of young ischemic stroke patients with hypertension (n = 30).

Clinical characteristics	n (%)
Sex	
Male	15 (50)
Female	15 (50)
Hypertension	30 (40)
Outcome at follow up	
Alive	28 (93)
Dead	2 (7)
mRS at follow up	
Favorable	20 (66.7)
Unfavorable	10 (33.3)
Clinical Characteristics	Mean±S.D.
Age (Years)	40±4.87
Duration of ischemic stroke before	2.68±0.94
NIHSS	9.28±6.84
GCS at admission	13.8±2.11
Duration of hospital stay (days)	5.74±6.07

Most of the young patients who presented to our hospital with ischemic stroke were in the age range of 36-45 years accounting for 24 (80%) of the total cases while patients less than 35 years contributed only to 6(20%) (Table 2).

**Table 2:** Age distribution of young ischemic stroke patients with hypertension (n = 30).

Age (years)	n (%)
16-25	0 (0)
26-35	6 (20)
36-45	24 (80)

In most of the patients with ischemic stroke, etiology was undetermined which accounted for 10 (33.3%) of the cases. Cardioembolic stroke was seen in 3 (10%) of patients. Cardiac causes of ischemic stroke identified were rheumatic heart disease, myocardial infarction, atrial fibrillation, and cardiomyopathy. Small vessel stroke (lacunar stroke) was also seen in 10 (33.3%) of patients. Large artery atherosclerosis as the cause of the ischemic stroke was observed in 4 (13.3%) of the patients. In the stroke of other determined etiology systemic lupus erythematosus (SLE), Tubercular meningitis associated vasculitis, CADASIL, reversible cerebral vasoconstriction syndrome, arterial dissection (Table 3).

**Table 3:** Etiology of Ischemic stroke in young patients with hypertension (n= 30).

Etiology	n (%)
Large artery atherosclerosis	4 (13.3)
Cardio embolic	3 (10)
Small vessel stroke (Lacunar stroke)	10 (33.3)
Stroke of other determined etiology	3 (10)
Stroke of undetermined etiology	10 (33.3)

In the present study, most of the patients who presented to hospital had moderate to severe disability at the time of admission and discharge. However, during follow up at 6 months favorable response (MRS 0 or 1) was seen in 20 (66.7%) of patients. Death during hospital stay or during follow up was observed in 2 (6.7%) of patients (Table 4).

**Table 4:** Modified Rankin Scale (mRS) at admission and follow up in young ischemic stroke patients with hypertension (n= 30).

mRS	At admission n (%)	Follow up at 6 months n (%)
0	2 (6.7)	13 (43)
1	2 (6.7)	7 (23.3)
2	2 (6.7)	6 (30)
3	13 (43)	2 (6.7)
4	9 (30)	0 (0)
5	0 (0)	0
6	2 (6.7)	2 (6.7)

## DISCUSSION

Hypertension was the most common risk factor for ischemic stroke present in our patients. Other important risk factors observed were the previous history of cardiac disease, excess alcohol consumption, hypercholesterolemia, smoking, diabetes mellitus, migraine, family history of stroke. The risk factor profile is similar to a study done by Lee, et al. in Southeast Asia with hypertension as one of the most common risk factors.^8^ Two or more two risk factors were observed in 35 (46.7%) of our patients.

Excessive alcohol abuse, including binge drinking, has been known to increase the risk of acute ischemic stroke in younger adults.^9,10^ Adverse effect on hemostasis, fibrinolysis, and blood clotting as well as increased risk of cardiac arrhythmias caused by alcohol may be the reason for the observed increased risk for ischemic stroke in alcoholics. Besides, alcoholics are also more likely to develop arterial dissection due to trauma.^11^

In our study, we evaluated prognosis of patients with ischemic stroke in young adults. During follow up at 6 months 20 (66.7%) of the patients with hypertension had favorable outcome with mRS score of 0 or 1. Death during hospital stay or during follow up was observed in 2 (7%) of patients with hypertension. According to MRS scale, during long-term follow up more than 70%-80% of the patients with ischemic stroke have significant improvement in functional status (mRS = 0-2), 10%-20% have moderate disability (mRS = 3) while about 10% are left with major disability after acute ischemic stroke (mRS score higher than 3).^12,4^

In our study ischemic stroke was more common in male than female. In the community and population based study, stroke was found to be more common in male than in woman particularly for those older than 35 years of age.^15,16^ Most of the participants in our study were older than 35 years of age which may have accounted for the difference in sex observed in our study.

In the present study the etiology of ischemic stroke due to undetermined causes and lacunar stroke constituted the largest group. Complete evaluation could not be carried out in all patients due either to unavailability of the test or lack of affordability of the patients. Cardio embolic stroke caused by rheumatic heart disease, atrial fibrillation, myocardial infarction and cardiomyopathy was next common etiological factor. Equally common to cardiac cause was small vessel stroke. In the stroke of other determined etiology arterial dissection, systemic lupus erythematosus (SLE), Tubercular meningitis associated vasculits, CADASIL, reversible cerebral vasoconstriction syndrome was identified.

In our study, we found lower NIHSS score during admission in patient with good functional outcome similar to earlier studies.^17,18^ Low GCS at admission was found in patients with worse outcome. Age of the patients, gender and duration of stroke before presenting to hospital, history of smoking, hypertension, diabetes mellitus and previous history of stroke or TIA did not influence outcome.

Since this study was conducted in a single centre, it may not reflect the outcome of patients with ischemic stroke of the whole country as facilities and access to hospital for the patients may vary by geography as Nepal is a hilly country with difficulty in reaching to hospital on time.

## CONCLUSIONS

We concluded from our study, hypertension is more common in the young adults with Ischaemic stroke as compared to other similar study. Hypertension is an important risk factor identified in young patients with ischemic stroke. Most of the patients have a good long term outcome while others are left with significant disability. Identification of the risk factors and their modification may help to take better care of those patients.
